# Isthmocele and Infertility

**DOI:** 10.3390/jcm13082192

**Published:** 2024-04-10

**Authors:** Giorgio Maria Baldini, Dario Lot, Antonio Malvasi, Doriana Di Nanni, Antonio Simone Laganà, Cecilia Angelucci, Andrea Tinelli, Domenico Baldini, Giuseppe Trojano

**Affiliations:** 1 MOMO’ FertiLIFE, IVF Clinic, 76011 Bisceglie, Italy; 2 Obstetrics and Gynecology Unit, Department of Biomedical Sciences and Human Oncology, University of Bari “Aldo Moro”, Piazza Giulio Cesare 11, 70124 Bari, Italy; antoniomalvasi@gmail.com; 3 Pathology Unit, Department of Precision and Regenerative Medicine and Ionian Area (DiMePRe-J), University of Bari “Aldo Moro”, 70125 Bari, Italy; 4 Unit of Obstetrics and Gynecology, “Paolo Giaccone” Hospital, Department of Health Promotion, Mother and Childcare, Internal Medicine and Medical Specialties (PROMISE), University of Palermo, 90127 Palermo, Italy; antoniosimone.lagana@unipa.it; 5 Gynecology and Obstetrics Clinic, University of Sassari, 07100 Sassari, Italy; c.angelucci@studenti.uniss.it; 6 Department of Gynaecology and Obstetrics, “Veris Delli Ponti” Hospital, and CERICSAL (Centro di RIcerca Clinico SALentino), “Veris delli Ponti Hospital”, 73020 Lecce, Italy; andrea.tinelli@unisalento.it; 7 Department of Maternal and Child, Gynecologic Oncology Unit, IRCCS Istituto Tumori “Giovanni Paolo II”, 70124 Bari, Italy; giuseppe.trojano@asmbasilicata.it; 8 Madonna Delle Grazie Hospital ASM, 75100 Matera, Italy

**Keywords:** isthmocele, cesarean scar defect, niche, infertility, IVF, ICSI, hysteroscopy

## Abstract

Isthmocele is a gynecological condition characterized by a disruption in the uterine scar, often associated with prior cesarean sections. This anatomical anomaly can be attributed to inadequate or insufficient healing of the uterine wall following a cesarean incision. It appears that isthmocele may impact a woman’s quality of life as well as her reproductive capacity. The incidence of isthmocele can range from 20% to 70% in women who have undergone a cesarean section. This review aims to sum up the current knowledge about the effect of isthmocele on fertility and the possible therapeutic strategies to achieve pregnancy. However, currently, there is not sufficiently robust evidence to indicate the need for surgical correction in all asymptomatic patients seeking fertility. In cases where surgical correction of isthmocele is deemed necessary, it is advisable to evaluate residual myometrial thickness (RMT). For patients with RMT >2.5–3 mm, hysteroscopy appears to be the technique of choice. In cases where the residual tissue is lower, recourse to laparotomic, laparoscopic, or vaginal approaches is warranted.

## 1. Introduction

Recently, a potential association between the presence of isthmocele and difficulty in conceiving has been reported [1]. This review aims to examine the relationship between isthmocele and conception difficulties specifically. In particular, the implication of this condition during assisted reproductive treatments has been analyzed. However, it is important to emphasize that, despite the existence of data supporting the hypothesis of a reduced likelihood of pregnancy in both natural conception and assisted reproductive techniques, there is currently a lack of sufficiently robust evidence to systematically recommend surgical intervention for isthmocele correction in patients desiring offspring.

Literature analysis indicates that the hysteroscopic approach appears to be the preferred treatment for isthmocele correction, although the residual thickness of the myometrial tissue must reach at least 2.5–3 mm [2]. This approach reflects a careful evaluation of technical and clinical aspects, aimed at enhancing reproductive success prospects in cases where surgical intervention is deemed necessary.

Isthmocele is a complex gynecological condition characterized by a disruption in the uterine scar, often associated with prior cesarean sections. This condition has only recently been identified as a distinct clinical entity and continues to garner interest and investigation among experts in the field [3]. Isthmocele is distinguished by the formation of a pouch or space in the lower part of the uterus, known as the isthmus, which can lead to the accumulation of blood and fluids in the uterine cavity. This anatomical anomaly can be attributed to inadequate or insufficient healing of the uterine wall following a cesarean incision. It is important to note that isthmocele can also occur after other uterine surgical procedures, such as myomectomy or resection of the uterine septum, surgical treatments for endometriosis, heterotopic pregnancies, or inflammations and infections of the uterus [4]. It appears that isthmocele may impact a woman’s quality of life as well as her reproductive capacity. It can affect the uterus’s ability to safely host a fetus during pregnancy or even impede pregnancy altogether [5].

## 2. Materials and Methods

In this review, we identified studies that describe or assess the isthmocele and its effect on female fertility.

We searched PubMed, Scopus, ResearchGate, Web of science and preprint archives for research articles published up to January 2024 using the following terms: “Isthmocele”, “Cesarean scar defect”, “uterine niche”, “Isthmocele & infertility”, “Cesarean scar defect & infertility”, “uterine niche & infertility”, “Cesarean scar defect repair”, “Isthmocele repair”, “isthmocele & IVF” and “Cesarean scar defect & IVF”.

Research titles were independently reviewed by four authors to eliminate studies that did not meet the issue before the full review of abstracts and full text of selected studies.

## 3. Epidemiology

The epidemiology of isthmocele has not been definitively estimated, as many cases are asymptomatic and therefore undiagnosed. A study conducted on women who had given birth via cesarean section found that 29% of the examined women had an isthmocele [6]. Another study reported that isthmocele was detected in over 32% of women who had undergone a cesarean section and subsequently developed symptoms such as painful menstruation or abnormal bleeding [7]. A further analysis published in 2020 concluded that the incidence of isthmocele can range from 20% to 70% in women who have undergone cesarean section [8] (Table 1).

A recent meta-analysis studied the association between cesarean scar defects (CSD) and abnormal uterine bleeding (AUB) [9]. The results revealed a significant association between the presence of isthmocele and an increased risk of AUB. Specifically, patients with isthmocele were found to have a 3.47 times higher relative risk of encountering episodes of abnormal uterine bleeding (AUB) in comparison to those without such defects. The prevalence of AUB in patients with isthmocele ranged from 25.5% in the general population of patients with at least one previous cesarean delivery, up to 76.4% in patients undergoing diagnostic examinations.

Overall, it appears that the incidence of isthmocele is on the rise in parallel with increasing cesarean section rates [10]. However, further research is necessary to fully comprehend the epidemiology of this condition.

## 4. Risk Factors

### 4.1. Patient-Related Factors

#### 4.1.1. Genetic Predispositions

Certain women may be born with congenital uterine anomalies, such as a thin uterine wall or an area of isthmocele agenesis, which increases the risk of developing an isthmocele [7]. Currently, there is no clear genetic predisposition for isthmocele development following a cesarean section. However, some genetic conditions can elevate the risk of complications during pregnancy and childbirth. Ehlers–Danlos syndrome and Marfan syndrome, genetic disorders of connective tissue, have been associated with isthmocele onset [11], as these conditions can affect the ability of uterine tissues to heal properly [12]. In these syndromes, connective tissue is weak and may be less capable of withstanding the stress caused by childbirth and subsequent healing processes [13]. Furthermore, weak connective tissues may be more susceptible to tears during a cesarean section, heightening the risk of isthmocele formation [14]. Certain genetic mutations affecting blood coagulation can increase the risk of postpartum hemorrhage, thereby facilitating isthmocele development [15]. Mutations in Factor V Leiden (FVL) and the Prothrombin (PT) gene mutation have been associated with an elevated risk of postpartum hemorrhage and an increased risk of isthmocele development [16]. Congenital hip dysplasia, a genetic disorder causing an anomaly in hip joint development, has also been correlated with isthmocele [17]. This may be attributed to the fact that the hip joint is directly connected to the pelvis and surrounding structures, thus any abnormality in the joint could impact the position of the uterus and the ability of the isthmic scar to heal properly [17].

#### 4.1.2. Gestational Diabetes

There is no clear correlation between gestational diabetes (GDM) and the development of isthmocele after a cesarean delivery. However, it may increase the risk of postpartum hemorrhage, which can be associated with the genesis of the pathology [18]. Additionally, gestational diabetes appears to be associated, like other forms of diabetes, with alterations in tissue healing and perfusion, thereby facilitating isthmocele formation [19]. Furthermore, GDM has been linked to reduced fibroblast activity, representing the cellular population responsible for scar tissue formation. This may potentially slow the healing of the uterine wall after cesarean section and elevate the risk of isthmocele formation [20].

#### 4.1.3. Endometriosis

Although isthmocele and endometriosis are distinct pathologies, some scientific evidence suggests a correlation between them [21]. Specifically, some women who have undergone a cesarean section and subsequently develop an isthmocele may have a slightly increased risk of developing endometriosis [22]. The precise mechanism underlying the correlation between isthmocele and endometriosis is not yet fully understood. However, several hypotheses have been proposed to explain this association. It appears that during a cesarean section, endometrial cells may migrate to the site of the isthmocele. This dissemination of endometrial cells could be facilitated by the surgical trauma and inflammation associated with the cesarean procedure [23]. This weakening may create an environment that can promote the implantation and proliferation of extrauterine endometriotic tissue [24]. Understanding the mechanisms underlying this connection necessitates further research and is of critical importance for the appropriate management and effective treatment of this complex clinical condition.

#### 4.1.4. Hypertension

A study published in 2018 proposed an association between hypertension and isthmocele [25]. The authors conducted a study on a group of women with isthmocele, comparing them to a control group without this condition. The results revealed that women with isthmocele exhibited a higher prevalence of hypertension compared to the control group. It is plausible that hypertension may induce compromised blood perfusion in the isthmocele region, promoting local alterations that facilitate the formation or persistence of the cavity in the uterine wall [9]. Furthermore, hypertension itself may represent a risk factor for the occurrence of scar adhesions in the uterine cavity, which could play a role in isthmocele development. Another study examined the correlation between gestational hypertension and isthmocele [26]. Researchers found that women with gestational hypertension had a slight increase in the risk of developing an isthmocele compared to women without this condition. Gestational hypertension could negatively impact the uterine wall healing process after a cesarean section, thereby promoting isthmocele formation. Vascular alteration and increased stress on uterine structures may contribute to the onset or persistence of isthmocele.

#### 4.1.5. Ectopic Pregnancy and Spontaneous Abortion

These complications can cause damage to the uterine wall, including the isthmus, which may increase the risk of developing an isthmocele [9]. Both isthmic ectopic pregnancies and spontaneous abortions are correlated with the onset of isthmocele. The precise mechanism through which these conditions contribute to isthmocele beginning is not yet fully understood. However, it is hypothesized that such events may trigger inflammatory processes and adhesion formation within the uterus, leading to abnormal thickening of the endometrium. Some researchers conducted a systematic review to examine the correlation between isthmocele and ectopic pregnancy [27]. Furthermore, the results suggest that in women who have undergone previous cesarean surgical intervention, screening for isthmocele during pregnancy may be advisable.

Another article had as its main objective to examine the risk factors for ectopic pregnancy in women. Specifically, the highest risk of ectopic pregnancy was observed in women who had undergone multiple cesarean sections. It was also highlighted that women with isthmocele may experience difficulties in conceiving and have a higher rate of spontaneous abortion compared to women without this uterine condition [28].

#### 4.1.6. High BMI and Lifestyle

Several studies have reported a correlation between body mass index (BMI) and the onset of isthmocele [29,30]. Additionally, a higher prevalence of intra-abdominal adhesion formation following a cesarean section has been observed in obese women [3]. Among the known risk factors for isthmocele development are the presence of multiple cesarean scars and inadequate healing of the uterine scar. Excessive body weight can apply pressure on the cesarean scar, thus contributing to isthmocele formation.

Cigarette smoking can also negatively influence the healing process and potentially contribute to isthmocele formation. Cigarettes contain a wide range of toxic chemicals that can have detrimental effects on the vascular system and compromise blood circulation. This leads to reduced perfusion and a diminished supply of oxygen and nutrients to the cells involved in wound healing, including the uterine scar. Some new articles published in 2021 [31] and in 2018 [32] highlighted a risk of developing isthmocele in women who smoke compared to non-smokers. It was postulated that cigarette consumption may thereby compromise uterine scar healing and negatively impact the scar tissue formation process.

#### 4.1.7. Retroflexed/Verted Uterus

A retroflexed or retroverted uterus is an anatomical condition in which the uterus exhibits a posterior angulation relative to the bladder. This position may be present from birth or acquired as a result of uterine pathologies such as fibroids, endometriosis, or pelvic adhesions [33]. The presence of a retroverted uterus does not inherently increase the risk of developing an isthmocele following a cesarean section.

However, studies investigated this potential relationship, albeit with conflicting results. The latter suggested that this anatomical condition might be associated with an increased risk of developing an isthmocele after cesarean section, as it may render the cesarean surgery more challenging, thereby elevating the risk of uterine injuries or other complications during the operation [34].

### 4.2. Factors Related to Delivery

#### 4.2.1. Cesarean Section Incision Level

The level of cesarean section incision can influence the risk of developing an isthmocele. Specifically, it appears that a transverse uterine incision is associated with a lower risk of developing an isthmocele compared to a vertical uterine incision [35]. This is because a longitudinal incision at the isthmus of the uterus involves greater muscular wall injury, potentially leading to weakness or defects in the muscular wall. A study examined women who had undergone a cesarean section and found that the risk of developing an isthmocele was significantly higher in women who had undergone a vertical incision compared to those with a transverse incision [36]. Another study found that the incidence of isthmocele was lower percentage in women who had undergone a transverse incision, compared to a higher percentage in women who had undergone a vertical incision [37].

#### 4.2.2. Uterine Suturing Technique

Uterine suturing techniques can influence the risk of developing an isthmocele after a cesarean section. Certain suturing techniques can lead to reduced perfusion in the isthmic region, creating an area of vulnerability conducive to isthmocele formation. Suturing techniques that may increase the risk of developing isthmocele include single-layer continuous suturing. This technique involves a single continuous suture to close the uterine incision. This technique appears to reduce blood flow at the isthmus and create an area of reduced uterine wall resistance [26]. Double-layer suturing involves closure in two layers before the myometrium and then the visceral peritoneum. This technique may increase blood flow at the isthmus and reduce the risk of isthmocele development [38]. Additionally, the correlation between isthmocele risk and the type of suture material used has been analyzed. Studies have demonstrated that the use of long-lasting absorbable sutures, such as poliglecaprone or polydioxanone thread, appears to reduce the risk of developing an isthmocele compared to the use of rapidly absorbable sutures [39,40]. A recent systematic review conducted by Genovese et al. [41] analyzed a total of six studies and concluded that closing the hysterotomy with a continuous double-layer suture technique would therefore be a suitable option to prevent the formation of cesarean scar defects. Specifically, the first layer should encompass the decidua, and the second layer should overlap the first.

#### 4.2.3. Adhesions

Pelvic adhesions are a common complication after a cesarean section and may be correlated with isthmocele. They result from the formation of scar tissue between pelvic organs, which can lead to symptoms such as pelvic pain, infertility, and difficulty in urination, or defecation [7]. The formation of pelvic adhesions can be influenced by various factors, including the type of uterine incision, the size of the uterus during pregnancy, the time elapsed since the cesarean section, or the presence of infections.

A study examined the correlation between pelvic adhesions and cesarean section. This found that 74% of women undergoing a cesarean section had pelvic adhesions during a subsequent surgical procedure [40]. Another study looked at the relationship between pelvic adhesions and the type of uterine incision. The study found that women undergoing a cesarean section with a transverse incision had a lower risk of developing pelvic adhesions compared to those undergoing a vertical incision [42]. Isthmocele appears to increase the risk of pelvic adhesion formation, and it seems associated with chronic inflammation in the isthmic zone and uterine cavity, which can lead to scar tissue formation [43,44]. Furthermore, pre-existing pelvic adhesions may influence the healing process of the isthmus after a cesarean section and increase the risk of its formation [2].

#### 4.2.4. Cervical Dilatation

Although the precise relationship between initial cervical dilatation and isthmocele onset remains unclear, several hypotheses offer a plausible explanation. It can be hypothesized that initial uterine dilation may exert greater pressure on uterine muscles and tissues, prolonging the period of stress they are subjected to [45]. This condition, in turn, would make them more susceptible to lacerations or weakening, facilitating isthmocele onset. Furthermore, early dilation might facilitate the entry of bacterial agents into the uterus, predisposing to infection, which, in turn, could further weaken uterine muscles and tissues, increasing the risk of developing an isthmocele [46].

This study [46] analyzed the correlation between the degree of cervical dilation at the time of cesarean section and the location of the uterine scar, which in turn correlated with the risk of developing isthmocele. In particular, the study highlighted patients undergoing cesarean section outside of labor or at early cervical dilation (<2 cm) that presented a uterine scar above the level of the internal uterine orifice, while emergent cesarean sections and those with advanced cervical dilation (>8 cm) were associated with the formation of uterine scars at the level or below the plane of the internal uterine orifice. Isthmocele formation was observed in 40% of patients with initial cervical dilation and 20% of patients undergoing cesarean section in advanced dilation. Therefore, early cervical dilation is considered a risk factor for isthmocele development.

#### 4.2.5. Ectopic Pregnancy on the Scar

The relationship between isthmic ectopic pregnancy and isthmocele has been recently studied [47,48]. Several researchers have examined this correlation to better understand the involvement of isthmocele in ectopic pregnancies. A study published correlated the presence of isthmocele with an increased risk of developing an ectopic pregnancy compared to those without this uterine condition [49]. The correlation between isthmocele and ectopic pregnancy in the group of women who had undergone uterine surgery was significantly higher among women with isthmocele than those without isthmocele. Isthmocele can represent a site for ectopic embryo implantation [4].

#### 4.2.6. Twin Pregnancy

During twin pregnancy, the uterus undergoes significant anatomical and physiological changes due to the presence of two fetuses [50]. This phenomenon leads to a considerable increase in the volume and weight of the organ, generating an increase in the mechanical load on the isthmic region of the uterus. The uterine isthmus, which represents the thinnest and most vulnerable portion of the uterine wall, is at particular risk of being influenced by this prolonged mechanical pressure. The theory of “mechanical load” suggests that the increase in volume and weight of the twin uterus exerts a compressive pressure on the isthmic region. This pressure could cause a series of pathological events, including the rupture or weakening of the uterine isthmus [51]. Consequently, an indentation or asymmetry in the uterine wall originating from the isthmocele may be formed. However, it should be emphasized that this hypothesis does not provide a comprehensive explanation for the onset of isthmocele in twin pregnancies. Other factors, such as previous uterine surgical interventions or individual anatomical characteristics, may interact with the mechanical load to influence the risk of developing an isthmocele.

## 5. Symptomatology

From a clinical perspective, isthmocele can have variable symptoms and complications. Some women may experience abnormal menstruation, such as heavy or irregular bleeding, while others may be asymptomatic. In some cases, isthmocele can cause dyspareunia (pain during sexual intercourse), chronic pelvic pain, or difficulty conceiving. The main obstetric complication is represented by an increased risk of uterine rupture [52].

### 5.1. Post-Menstrual Spotting

Post-menstrual spotting refers to the appearance of light and short-duration vaginal discharge immediately after the end of the menstrual period [53]. Isthmocele, by creating a sac in the uterine cavity, can accumulate blood during menstruation. When menstrual flow decreases, the accumulated blood can exit the uterine cavity through the isthmocele, causing post-menstrual spotting [54].

### 5.2. Prolonged Bleeding

Isthmocele can be the cause of chronic inflammation in the surrounding area, leading to episodes of prolonged bleeding. The cavity may interfere with uterine contraction and the formation of blood clots after delivery. According to a recent study [55] among women with isthmoceles, the prevalence of postmenstrual spotting was 20.0%, while in women without isthmoceles, it was 8.3%. This indicates that women with isthmoceles were more likely to experience postmenstrual spotting. The prevalence of postmenstrual spotting was even higher in the subgroup of women with large isthmoceles, where it reached 25.9%, while women without isthmoceles had a prevalence of 9.5%.

Chronic inflammation associated with isthmocele can therefore cause an increase in uterine bleeding and prolong its duration after childbirth. The correlation between isthmocele and post-menopausal bleeding was evaluated in some other publications [56,57,58]. These studies included women with an average age of 49 years who had experienced post-menopausal bleeding. Isthmocele was found in more than 30% of the women. According to the findings, isthmocele could be a risk factor for post-menopausal bleeding.

### 5.3. Intermittent Spotting

The scar tissue generated by isthmocele can create an indentation in the uterine wall, where menstrual blood can accumulate. This can prevent blood from being expelled regularly during menstrual flow, causing intermittent blood loss [59]. Additionally, scar tissue can interfere with the normal contraction of the uterus during the menstrual cycle. Contractions are crucial for expelling menstrual blood, and any factor that interferes with these contractions can be a source of irregularities [60].

### 5.4. Pain

The pressure exerted by the isthmocele on the surrounding tissues can cause pain, especially during sexual intercourse or physical activity. Dyspareunia can be a common symptom associated with isthmocele [61]. In a sample of women with pelvic pain, it was found that a great majority of women with isthmocele also had dyspareunia. This suggests that the presence of scar tissue inside the isthmocele can irritate the surrounding tissues and pain during sexual intercourse. Other authors evaluated the presence of dyspareunia and other symptoms in women with isthmocele like chronic pelvic pain [62].

### 5.5. Dysfunctional Bladder

Dysfunctional bladder and isthmocele can be correlated due to their anatomical proximity and the possible interference between them. Dysfunctional bladder refers to a condition in which the bladder does not function properly, causing symptoms such as urinary incontinence, difficulty urinating, increased urinary frequency, or pelvic pain [63]. Since the bladder is located near the uterus and isthmocele, a significant isthmocele can exert pressure on the bladder or interfere with its normal function [63]. For example, a large isthmic sac can press on the bladder, causing a narrowing of the space available for urine accumulation. This can lead to increased urinary frequency or more frequent urination. In some cases, isthmocele can also compress the urethra, causing difficulty urinating or problems with bladder emptying [64]. Furthermore, both isthmocele and bladder dysfunction can be a consequence of a previous uterine surgical intervention.

### 5.6. Obstetric Complications in Future Pregnancies

Isthmocele can increase the risk of uterine rupture during labor. Since isthmocele represents an area of weakness in the uterine wall, intense uterine contractions could cause the rupture of the uterine wall in that specific area [65]. Additionally, isthmocele can influence the position and attachment of the placenta. This increases the risk of complications such as placenta previa, a condition where the placenta partially or completely covers the internal uterine opening. Placenta previa is associated with an increased risk of bleeding during pregnancy and childbirth [66]. Some studies suggest [67] that isthmocele may contribute to the onset of placenta accreta, a condition where the placenta abnormally attaches to the uterine wall, increasing the risk of severe bleeding and the need for surgical interventions. The presence of an isthmocele can also influence the type of fetal presentation. A large isthmocele could hinder the correct positioning of the fetus in the uterus, increasing the likelihood of abnormal presentations, such as breech presentation or transverse presentation [67]. These abnormal conditions can be associated with the use of operative delivery or cesarean section.

## 6. Infertility and Isthmocele

### 6.1. Infertility

The presence of anomalies in the uterine scar after cesarean section or gynecological surgery appears to be associated with reduced fertility. According to the literature, the risk of infertility in women with isthmocele is 4–19% [68,69,70]. However, the exact mechanism leading to the condition of subfertility and infertility has not yet been defined. Isthmocele itself, local inflammation, the accumulation of fluid (hydrometra) at the isthmocele level, or other unknown factors have been proposed as possible pathogenetic mechanisms [71].

A proposed mechanism focuses on the flow of blood or bleeding from the isthmocele into the uterine cavity and/or vagina leading to an excess of iron (for hemoglobin degradation) with a cytotoxic effect on the embryo [45] or determining impairment of the endometrial receptivity and uterine microbiota, afflicting the implantation [72,73,74]. Among the various proposed mechanisms to explain this evidence, it is believed that isthmocele may be associated with the occurrence of hydrometra, a condition characterized by the accumulation of fluid in the uterine cavity.

A recent study examined the impact of isthmocele on the endometrium in the presence or absence of hydrometra. A total of 141 women with a diagnosis of infertility, prior cesarean section, and large isthmocele defined by residual myometrial thickness less than 3 mm were recruited. The evaluation of endometrial thickness did not show statistically significant differences in the presence or absence of endocavitary fluid; however, a bias may be represented by the timing of recruitment. The patients were recruited at various phases of the menstrual cycle without correction for this variable. Therefore, further studies are needed to identify the most opportune time during the menstrual cycle for endometrial evaluation [75]. A retrospective study conducted on 1793 patients undergoing assisted reproductive treatments showed a lower pregnancy rate in women with previous cesarean section compared to women with prior vaginal delivery and an even lower pregnancy rate was observed in patients with isthmocele [76]. It is believed that the accumulation of endocavitary fluid may reduce the embryo implantation rate, likely due to the embryotoxic effect of the degradation products of hemoglobin [77]. Literature analysis shows that various mechanisms have been proposed to explain how the collection of endouterine fluid (hydrometra) associated with isthmocele may interfere with the embryo implantation process in uterine tissue [78,79]. Previous studies have demonstrated that surgical intervention to correct defects in the cesarean scar can effectively restore uterine anatomy to a normal condition, thus preventing the accumulation of fluid within the endometrial cavity [80].

If the isthmocele is large enough and located along the natural pathway of sperm towards the uterus, it can obstruct their passage, thus hindering their ability to reach the uterus and consequently fertilize the egg. Additionally, the isthmocele can create an inflammatory environment in the surrounding area, which damages the sperm and reduces their motility. The presence of mucus or scar tissue within the isthmocele can further alter the uterine environment, making it more challenging for sperm to survive and move effectively. These factors collectively diminish the chances of conception for a couple seeking to conceive, especially if the isthmocele is substantial and remains untreated [24,81].

During the years, several studies were conducted to assess the possibility of fertility restoration in women with isthmocele after surgical repair, some of them are sorted in Table 2.

A recent systematic review [85] incorporating thirteen studies, comprising a randomized controlled trial, six prospective case series, and six retrospective case series, scrutinized surgical interventions for isthmocele-associated secondary infertility in a cohort of 234 patients. Within this cohort, 188 individuals underwent intervention via hysteroscopy, thirty-six through laparoscopy, seven via laparotomy, and three through a vaginal approach. Collectively, 153 patients (65.4%) attained pregnancy, with the randomized controlled trial registering a 75% pregnancy rate for hysteroscopy in contrast to 32% for untreated cases. Among the documented pregnancies, a noteworthy 87.1% (101 out of 116) culminated in live births. Adverse events, encompassing the necessity for reoperation, were infrequent at a mere 2%. The results suggest that surgical amelioration of isthmocele, notably through hysteroscopy, with a residual myometrial thickness of no less than 2.5 mm, may offer an efficacious recourse for addressing isthmocele-linked secondary infertility, coupled with a relatively modest incidence of complications. A retrospective study from 2019 [5] analyzed a population of 35 patients diagnosed with isthmocele randomized into two groups based on the ability to achieve pregnancy in the 12 months following the diagnosis of isthmocele. Infertile patients underwent surgical correction of the isthmocele with a pregnancy rate of 56.3% one year after treatment. Patients who did not achieve a restoration of fertility after surgical treatment showed older age, higher BMI, and multiple prior cesarean sections with larger isthmoceles. However, the retrospective nature and the small number of patients involved in the study represent a limitation of this work. The study conducted by Harjee [86] investigated the relationship between isthmocele and recurrent pregnancy loss (RPL). RPL is defined as the presence of at least two consecutive pregnancy losses within the first 20 weeks of gestation. The study results highlighted that women affected by isthmocele were more likely to experience RPL compared to women without this condition. Furthermore, it was observed that the size of the isthmocele was also correlated with the risk of miscarriage. Women with larger isthmoceles showed a higher risk of recurrent pregnancy loss compared to those with smaller ones. In 2020, Cohen SB et al. conducted a retrospective cohort study on 39 patients with a symptomatic niche and secondary infertility treated by hysteroscopic isthmocele resection. One year after the hysteroscopic resection, eighteen patients conceived (fourteen spontaneously and four following IVF), leading to a cumulative pregnancy rate of 46.15%, suggesting that Hysteroscopic niche resection should be considered an effective treatment in patients suffering from secondary infertility [83]. Yang G et al. in 2023 conducted a prospective observational study to evaluate the clinical effectiveness and pregnancy rate after hysteroscopic resection (HR) and/or vaginal repair (VR) in patients with isthmocele. During the study, 41 patients who wanted to conceive became pregnant with a median pregnancy time of 22 months after VR and 12 months after HR. Among patients with subsequent infertility, 31.6% achieved pregnancy by unassisted mode and 29.8% by IVF/ICSI. Moreover, among patients with previously failed IVF/ICSI treatment, 60% (12/20) obtained pregnancy, including 71.4% (10/14) after HR and 33.3% (2/6) after VR. Their results suggested that surgical intervention could improve the clinical pregnancy rate of patients with isthmocele [84].

### 6.2. IVF and Isthmocele

Recently, it has been hypothesized that isthmocele may alter the outcomes of assisted reproductive technology (ART) treatments. Isthmocele can have a significant impact on the uterus’s ability to provide a favorable environment for embryonic implantation. This defect in the uterine wall can create an area of compromised blood supply and nutrients necessary for proper embryo development. This may negatively affect the embryo’s ability to grow properly within the uterine tissue. Additionally, the association between the presence of an isthmocele and the outcomes of ART treatments has been examined [82]. Various research teams have involved a group of women undergoing in vitro fertilization (IVF), showing a lower pregnancy rate in the group of patients with isthmocele compared to patients without this condition. Furthermore, the rate of spontaneous abortion was found to be increased in the group with isthmocele compared to the control group. These results suggest a negative correlation between the presence of an isthmocele and ART outcomes. A 2023 study examined the relationship between isthmocele size and ART outcomes [87]. The study demonstrated that women with a larger isthmocele have reduced implantation rates and an increase in complications during IVF treatment. Specifically, there was an increase in the incidence of spontaneous abortion and a lower rate of ongoing pregnancies in the group with a larger isthmocele. These results suggest that isthmocele size can significantly influence implantation capacity and embryo survival. Isthmocele may alter uterine contractility, compromising its ability to provide an optimal environment for implantation and embryonic development [88]. Some researchers have also suggested that isthmocele may be associated with uterine inflammation, creating an unfavorable environment for embryonic implantation [89]. This inflammation may arise from the accumulation of fluids or residues in the isthmocele cavity, creating a condition conducive to bacterial or infectious proliferation. Inflammation may then interfere with the interaction between the embryo and the endometrium, compromising the implantation process. A prospective observational study [90] conducted on a population of women with prior cesarean section and isthmocele undergoing ART with controlled ovarian stimulation showed that 40% of patients exhibited an accumulation of endocavitary fluid with adverse outcomes after euploid embryo transfer. The primary risk factor for hydrometra during hormonal stimulation was represented by isthmocele size; additionally, the prior number of cesarean sections correlated positively with increased fluid accumulation in the uterine cavity during treatment. Moreover, isthmocele size itself showed an increase during stimulation treatment with the accumulation of larger quantities of endouterine fluid. In 2002, the Chien working group analyzed the pregnancy rate in patients with hydrometra and tubal or non-tubal infertility, showing a reduced implantation rate in the presence of hydrometra during ART treatment and an absence of pregnancy when embryo transfer was performed in the presence of endouterine fluid [91]. A cutoff of 3.5 mm of endocavitary fluid was also identified beyond which the embryonic implantation capacity would be null [92].

In 2023, Mensi L. et al. retrospectively analyzed a cohort of 114 patients undergone IVF for secondary infertility to see how common Cesarean scar defects among them was. A total of 76 patients were diagnosed with isthmocele, with a prevalence of 67% (95% CI 58 to 75%). The clinical pregnancy rate (adjusted OR 0.31, 95% CI 0.13 to 0.72) and live birth rate (adjusted OR 038, 95% CI 0.17 to 0.86) were significantly lower among affected women [93].

Another retrospective study by Diao J. et al. investigated the effect of Cesarean scar defect on pregnancy outcomes after IVF. The study included 401 women with a previous vaginal delivery (VD), and 433 women with a history of delivery by caesarean section, among whom 359 had a caesarean scar (CS) without a defect and 74 had a caesarean section defect (CSD). All the patients had IVF/ICSI treatment.

Although they found no statistically significant differences in biochemical pregnancy rate, live birth rate, clinical pregnancy rate, mean implantation rate, or abnormal pregnancy rate between the CS and VD groups, the live birth rate and mean implantation rate in the CSD group were found to be statistically significantly lower than those in the VD group (21.6 vs. 36.4%, adjusted OR 0.50 [0.27–0.9]; 0.25 ± 0.39 vs. 0.35 ± 0.41, adjusted OR 0.90 [0.81–0.99]). In the subgroup of women aged ≤35 years, in the CSD group the live birth rate, biochemical pregnancy rate, clinical pregnancy rate, and mean implantation rate were all significantly lower than those in the VD group (21.4 vs. 45.8%, adjusted OR 0.35 [0.15~0.85]; 38.1 vs. 59.8%, adjusted OR 0.52 [0.24–0.82]; 31.0 vs. 55.6%, adjusted OR 0.43 [0.19–0.92]; 0.27 ± 0.43 vs. 0.43 ± 0.43, adjusted OR 0.85 [0.43 ± 0.43]). However, for women over 35 years, no statistically significant differences were found in any pregnancy outcome among the three groups. Their results suggested that CS without a defect does not decrease the live birth rate after IVF or ICSI compared with a previous VD, while the presence of a CSD in especially young women (age ≤ 35 years), significantly reduced the chances of obtaining a subsequent pregnancy [94]. In 2022, our group assessed the outcomes of two groups of isthmocele-afflicted patients undergoing in vitro fertilization (IVF): women undergoing blastocyst-stage embryo transfer on the fifth day compared to women undergoing transfer on the third day. The results demonstrated that the risk of ectopic pregnancy on the cesarean scar was significantly lower in the group of patients who performed embryo transfer on the fifth day. According to our results, when isthmocele is diagnosed, transferring the embryo on Day 5 at the blastocyst stage appears to minimize the risk of pregnancy implantation it [95]. According to our hypothesis, this discrepancy can be attributed to the fact that embryos transferred on the fifth day are generally in a more advanced stage of development, which reduces the probability of migrating onto the uterine scar. In 2023, a retrospective cohort study by Yao W. et al. investigated the relationship between caesarean scar defect and reproductive outcomes of in vitro fertilization (IVF) and intracytoplasmic sperm injection (ICSI). They included 2231 women who had undergone 2515 IVF cycles. They found a reduced live birth rate between women with niche and women without a niche (18.99% vs. 31.51%, 0.51, 95% CI: 0.34–0.77), reduced positive human chorionic gonadotropin test rate (34.08% vs. 46.40%, adjusted odds ratio [aOR]: 0.61, 95% confidence interval [CI]: 0.43–0.87), reduced clinical pregnancy rate (29.05% vs. 42.25%, aOR: 0.57, 95% CI: 0.39–0.82) and reduced implantation rate (25.87% vs. 36.95%, aOR: 0.53, 95% CI: 0.38–0.76). They also found that the niche group had a 7.28% to 18.22% increase in miscarriage rate even though there was no statistical significancy [96]. In 2023, Vitagliano et al. executed a systematic review and meta-analysis on the effect of isthmocele in IVF/ICSI treatment. They included eight studies and demonstrated with a moderate quality of evidence (Grading of Recommendations Assessment, Development and Evaluation grade ¾) the negative effect of isthmocele on LBR in women undergoing IVF. Their results confirmed that it is not the Cesarean section per se that impairs the fertility, but the presence of the defect. They also demonstrated that the intracavity fluid accumulation in isthmocele before embryo transfer impairs the outcome of the treatment [97].

### 6.3. Isthmocele and Ovarian Stimulation

Currently, there is no dedicated ovarian stimulation strategy exclusively for these patients, although this requires further investigation and exploration. The ovarian stimulation protocol adopted follows standard clinical practice guidelines and utilizes both GnRH agonist and GnRH antagonist protocols [98].

## 7. Diagnosis

The diagnosis of isthmocele primarily relies on ultrasound examination [99,100], which enables visualization of the defect in the uterine wall and assessment of the dimensions and depth of the isthmocele. In more complex situations, more advanced diagnostic techniques, such as magnetic resonance imaging, may be necessary to obtain a detailed evaluation. Also, hysterosalpingography or saline infusion sonohysterography (SIS) can be used to study this defect of the anterior wall of the uterine isthmus. This imaging technique allows for the identification of the presence of a cavity or deformity in the uterine wall, indicative of the presence of an isthmocele. In some cases, hysteroscopy may be necessary. Performing an MRI can confirm the presence and size of the isthmocele, as well as provide valuable information for evaluating any associated complications.

Pelvic transvaginal ultrasonography (with or without SIS) and MRI are the principal diagnostic techniques because allow to evaluation of the thickness of the remaining myometrium which is the most important parameter of assessment of the isthmocele [101,102].

### 7.1. Transvaginal Ultrasound Examination

From an ultrasound perspective, isthmocele may exhibit specific characteristics that aid in its identification and assessment. During a transvaginal ultrasound, the isthmocele may be visualized as a hollow cavity or a sac-like area in the uterine wall, generally in the isthmic region. The ultrasound image of the isthmocele may show an area of weakness or thinness in the uterine wall, with a separation between the anterior and posterior walls of the uterus. The size and shape of the isthmocele may vary from case to case, but it is generally observable as a visible cavity within the uterus. Additionally, ultrasound parameters can be used to measure the dimensions of the isthmocele, such as length, width, and depth of the cavity. The ultrasonographic criteria for diagnosis of the Isthmocele and its classification were proposed by Jordans et al. in 2019, whose group modified the Delphi procedure. Isthmocele was defined as an indentation with a depth > 2 mm at the site of the Cesarian scar, and it was subclassified as simple; simple with one branch; complex (with >1 branch) [103].

A small isthmocele may be defined as a cavity with a length of less than 1 cm and a width of less than 1.5 cm. However, a large isthmocele may have a length exceeding 1.5 cm and a width that can extend up to 3 cm or more.

**Figure 1:**
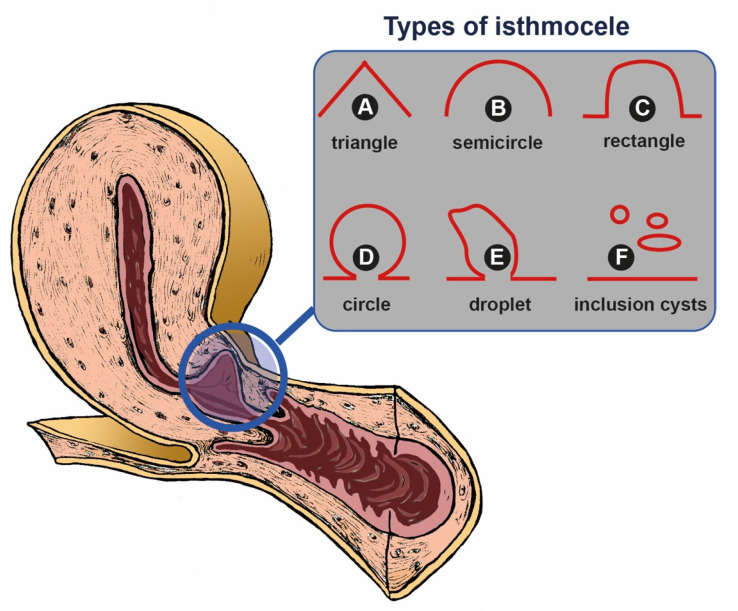
The picture shows a drawing of a longitudinal section of Uterus, Cervix and Vagina. In the blue circle in correspondence to the uterine isthmus, there is an enlargement (a sack or pouch) of the uterine cavity: isthmocele. On the top right of the image, there is a schematic representation of the possible different shapes of isthmocele: (**A**) triangle shape; (**B**) semicircle shape; (**C**) rectangle shape; (**D**) circle shape; (**E**) droplet shape; (**F**) inclusion cysts.

Bij de Vaate et al. proposed a classification for the assessment of the isthmocele (Figure 1). According to this classification, it is possible to distinguish types of isthmocele based on the shape: triangle (Figure 1A), semicircle (Figure 1B), rectangle (Figure 1C), circle (Figure 1D), droplet (Figure 1E) and inclusion cyst (Figure 1F) [65,104].

Other important parameters to evaluate for a correct assessment of the isthmocele are: Residual myometrial thickness (RMT), isthmocele depth (DI), isthmocele width (WI), cervical thickness (CT), distance from the fundus to isthmocele (DFUI) and distance from the isthmocele to the cervix (DCI) [105,106].

The RMT is the shortest distance between the endometrium at the level of the scar and the uterine serous surface taken on a longitudinal scan. RMT, length, depth should be measured on a sagittal scan, and the width on a transverse scan. Color Doppler could be used to differentiate isthmocele from adenomyomas, adenomyosis, and hematomas.

It is important to emphasize that isthmocele size is not the sole determining factor for symptom severity or potential associated complications. Other factors, such as the presence of endometriosis, inflammation, or association with infertility, can influence the clinical presentation and management of isthmocele.

There are two other classifications:

1. Gubbini et al. [79] suggested a classification based on the surface area (A) of the isthmocele, considering the shape of the defect as an isosceles triangle to calculate the surface area using the formula: (Base × height)/2. Isthmoceles were classified into three grades: grade 1, A < 15 mm^2^; grade 2, 16 mm^2^ < A < 25 mm^2^, and grade 3 A > 25 mm^2^;
2. Ofili-Yebovi D et al. [107] classified isthmoceles based on myometrial thinning at the site of defect. They calculated the ratio between the myometrial thickness at the level of the defect and the thickness of the adjacent myometrium. A severe defect was defined as a ratio >50% and dehiscence as a ratio equal or superior to 80%.

### 7.2. Magnetic Resonance Imaging

MRI allows us to explore the pelvic cavity and to obtain an accurate evaluation of the isthmocele. Indeed RMT, length, depth, width, adjacent myometrial thickness, and isthmocele–vescicovaginal fold can all be measured on sagittal and transverse planes, and MRI also allows to identification the presence of residual menstrual blood as hypersignal spots on T1-weighted images. After the microscopic evaluation of isthmocele evaluated both with RMT and TVS the differences between the measurements were not statistically significant [8]. MRI provides a clearer view of the defect before surgery.

### 7.3. Hysteroscopy

From a hysteroscopic point of view, isthomecele appears as a cavity on the anterior side of the isthmus. With the hysteroscopic approach is also possible to visualize hypervascularized areas and dendritic vessels with hemorrhage or polyps [66,100], which could suggest bleeding from the defect; it can also allow to find endometriotic lesions on this site.

## 8. Treatment

### 8.1. Medical Treatment

In cases of asymptomatic or minimally impactful isthmocele on fertility, a monitoring approach without active therapeutic intervention can be adopted [100]. In some cases, isthmocele may spontaneously resolve over time [108]. The choice of treatment will be influenced by patient peculiarities, isthmocele size and location, and other relevant clinical considerations.

#### 8.1.1. Estroprogestins

Combined oral contraceptive pills containing synthetic estrogens and progestins are often considered in the context of isthmocele for managing associated symptoms [109]. This hormonal therapy can offer significant benefits in reducing abnormal bleeding, stabilizing the uterine lining, and regulating the menstrual cycle. The primary goal of using oral contraceptive pills in isthmocele treatment is to manage symptoms and improve the quality of life for patients affected by this condition. The estrogens in oral contraceptive pills help maintain integrity, reducing the incidence of abnormal bleeding associated with isthmocele. Additionally, the presence of synthetic progestin further stabilizes the endometrium, preventing excessive thickening or alterations that may exacerbate symptoms.

#### 8.1.2. GnRH Analogues

Gonadotropin Releasing Hormone (GnRH) analogues in isthmocele treatment, by regulating the release of gonadotropic hormones such as follicle-stimulating hormone (FSH) and luteinizing hormone (LH), affect the production of estrogen and progesterone. GnRH analogues can be administered through various routes, including injections, nasal sprays, or subcutaneous implants. Their action involves a temporary “suspension” of ovarian activity, reducing estrogen synthesis and suppressing ovulation. This intervention aims to mitigate abnormal bleeding and stabilize the uterine lining [110]. The use of GnRH analogues in isthmocele treatment is primarily considered in circumstances where more incisive hormonal control is required than with oral contraceptive pills, to manage symptoms more effectively.

#### 8.1.3. Progesterone-Releasing IUDs

Progesterone-releasing intrauterine devices (IUDs), characterized by their T-shaped form and placement within the uterus, have the peculiarity of gradually and continuously releasing synthetic progesterone. While widely used as a contraceptive method, these IUDs can also be a therapeutic perspective to consider for isthmocele treatment, to mitigate symptoms related to abnormal bleeding and menstrual cycle irregularity. Synthetic progesterone inhibits ovulation. This regulates the menstrual cycle and reduces the risk of bleeding and hormonal fluctuations associated with isthmocele [111]. Additionally, it modifies the thickness of the endometrium, reducing hypertrophy and thickening. This contributes to the prevention of bleeding and endometrial stabilization, alleviating isthmocele-related symptoms. Treatment with GnRH agonists (gonadotropin-releasing hormone agonists) is associated with clinical improvement of pelvic pain, dysmenorrhea, and dyspareunia associated with endometriosis and isthmocele.

#### 8.1.4. Probiotics

Histological analysis revealed the presence of chronic infiltrative inflammation in most patients with isthmocele. The accumulation of erythrocytes and fluids within the isthmocele may be correlated with infections and chronic endometritis. This inflammatory process may show similarities with hydrosalpinx and endometriosis. Research on the endometrial microbiota has highlighted high concentrations of *Escherichia coli* in the menstrual flow of patients affected by endometriosis. Furthermore, a significant increase in bacteria belonging to the families Streptococcaceae and Staphylococcaceae was observed in the cystic fluid of women with isthmocele compared to control subjects. In this regard, the use of GnRH agonists for at least three months appears to reduce the accumulation of blood in the isthmocele and scar tissue [109,112].

### 8.2. Surgical Approaches

The therapeutic approach depends on the symptoms and severity of the condition [112]. In asymptomatic or mild cases, periodic monitoring without active medical intervention may be sufficient. However, if symptoms are significant or interfere with the woman’s quality of life, corrective surgical intervention may be necessary. Surgical intervention for isthmocele can involve various options, such as resection or excision of scar tissue, suturing of the uterine defect, or a transvaginal approach involving the creation of a bridge or membrane to reinforce the uterine wall. In 2020 Donnez proposed an algorithm for treatment of the isthmocele [8] based essentially on the wish to conceive/infertility, symptoms and RMT.

#### 8.2.1. Hysteroscopic Approach

Hysteroscopy is the treatment of choice for isthmocele. This procedure allows for direct examination and treatment of the isthmocele. During hysteroscopy, it is possible to proceed with the resection of the upper and lower margins of the isthmocele and with the ablation of its cavity [35,109]. This technique allows symptomatic women to be free from symptoms related to isthmocele. Hysteroscopic reshaping involves flattening the endometrial tissue inside the isthmocele to prevent the accumulation of blood or menstrual residues and can be used in women with residual myometrial thickness (>3 mm) for whom isthmocele resection can be deferred. The advantage of hysteroscopy over surgery is its minimally invasive approach. However, laparoscopic correction is the preferred method in women with RMT < 2.5 mm and a desire for future pregnancies. It can sometimes be difficult to precisely locate the isthmocele, which is why a combination of hysteroscopy with transillumination is preferred to accurately identify the isthmocele.

In a study [113], it was found that in women who are unclear about their future needs, comparing medical therapy with hysteroscopic procedures showed that hysteroscopic resection may be the treatment of choice because it is minimally invasive and produces good therapeutic results. In patients with a history of infertility, ectopic pregnancy, more severe isthmocele, lower parity, and fewer cesarean sections, laparoscopic isthmoplasty is preferred over the hysteroscopic approach. Both methods have similar effects on mid-cycle vaginal bleeding, duration of post-menstrual spotting, and pain. However, it appears that hysteroscopic treatment may be associated with a higher risk of dyspareunia and dysmenorrhea [114].

#### 8.2.2. Laparoscopic Surgical Approach

In some situations, especially when isthmocele is associated with a reduction in myometrial thickness, laparoscopic surgery may be indicated [115]. This procedure is performed by allowing the surgeon to access the isthmocele and reinforce the anterior wall of the uterus. This method is preferred for larger defects to avoid subsequent complications such as uterine rupture [116]. Nezhat et al. demonstrated that a two-layer repair without passage inside the uterine cavity can reduce symptoms in 77% of patients, restore fertility in 73% of patients, and reduce time to conception [39]. The combined use of hysteroscopy without perfusion in the context of operative laparoscopy allows for observation of the uterine lumen without the use of a backflow fluid because pneumoperitoneal gas fills the uterine lumen. Intraoperative monitoring by hysteroscope and laparoscope allows for visualization of the lesion site from both sides during resection. This procedure allows for precise identification of the lesion area, complete removal of the lesions, and prevention of excessive resections that may reduce uterine function and increase perinatal risk [117]. Fluorescence-guided laparoscopic niche detection represents a new approach that may help prevent bladder injuries and unnecessary tissue preparation [118,119].

#### 8.2.3. Robot-Assisted Surgery Approach

Robot-assisted surgery is a similar option to laparoscopic surgery but employs a robotic system to perform the procedures. This approach may offer the surgeon greater precision and maneuverability during the intervention [120].

#### 8.2.4. Suturing Technique

A double-layer approach is preferred, i.e., one layer for two-thirds of the myometrium and the second layer for the remaining one-third of the myometrium and the serosa. Laparoscopy allows for a greater increase in RMT thickness during follow-up compared to hysteroscopy [41].

#### 8.2.5. Transvaginal Surgical Approach

The transvaginal approach appears to be a feasible, effective, and safe modality to repair the uterine defect and restore the original myometrial thickness. It is a minimally invasive procedure, scarless, and cost-effective. It ensures rapid recovery and a relatively painless postoperative course with a quick return to normal function. Kaya C. et al. proposed a transvaginal approach guided by hysteroscopy in women with multiple cesarean sections [121]. It has proven to be an advantageous technique, especially for shorter hospital stays and shorter surgery durations compared to operative laparoscopy [122]. The effectiveness of transvaginal repair is comparable to laparoscopic repair and may be a more convenient and cost-effective surgical approach in managing patients with post-cesarean section uterine wall defects.

## 9. Histopathologic Approach

Isthmocele is not a very common finding in surgical pathology. However, it is generally identified in hysterectomy and isthmoplasty specimens. The histologic aspects of this pathology are not widely described; despite this, the pathologist has an influential role to correlate the microscopic findings with symptoms, levels, completeness of resection, previous procedures, risk of thinning of the base and future pregnancy rupture [123]. The macroscopic examination is very heterogeneous depending on the type of surgical specimen. Histologically, a pathologist looks for many criteria, especially the types of the lining mucosa, luminal contents, the edge wall stroma, the underlying residual myometrial tissue, the scar area and fibrofatty tissue, and the base of the pouch. Delving into the first two aspects, the type of the lining mucosa of the edges may correspond to the level from the internal cervical os. Specifically, endometrial-isthmic lined edges are usually high-intermediate situated isthmoceles, whereas endocervical–isthmoendocervical ones are low-situated. This important difference is associated with specific symptoms. The edge wall stroma is composed by disorganized fibromuscular mesenchymal tissue with thick blood vessels and tortuous nerve bundles [124]. Exceptionally, it is possible to also find hemorrhage, cysts, fibroblastic stroma, metaplasia, epithelial atypia, inflammation, foreign body giant cell reaction, calcifications, adenomyosis, scar endometriosis, polyp, leiomyoma [124].

Several authors during decades studied the innervation of the cervix the isthmic region, among them, a group led by Malvasi A. [125,126] described the neurofibers and neuropeptides/neurotransmitters of the cesarean scar. In 2010, they investigated the substance P (SP) and vasoactive intestinal peptide (VIP) neurotransmitters and neurofibers on the caesarean scar of in labor patients. They found higher SP levels in repeat CS, while the VIP levels were reduced. They proposed that the increase of SP could be linked to the attempt to achieve cervical ripening in a post-CS cervix. The decrease of VIP could affect the relaxation of the internal uterine orifice, compromising the LUS formation and cervical ripening [126].

## 10. Discussion

Isthmocele is a recognized complication that can occur in women who have undergone previous cesarean sections. These defects can lead to various symptoms such as spotting, dysmenorrhea, pelvic pain, and infertility. Therefore, healthcare providers must be vigilant in suspecting CSDs in women presenting with these symptoms, especially those with a history of previous cesarean sections.

Transvaginal ultrasound (TVS) with adequate measurements is considered a key diagnostic tool in identifying CSDs. TVS allows for detailed visualization of the cesarean scar area, enabling healthcare providers to assess the presence and severity of any defects. However, the role of magnetic resonance imaging (MRI) in the diagnosis of CSDs should also be considered. MRI offers the advantage of providing detailed imaging without being operator-reliant, potentially offering additional information that may not be as easily obtained through TVS alone.

Numerous studies have examined the impact of isthmocele on fertility, evaluating the relationship between CSD and secondary infertility with recourse to ART; however, currently, there is not sufficiently robust evidence to indicate the need for surgical correction in all asymptomatic patients seeking fertility [2].

Studies have shown that women with isthmocele may experience reduced pregnancy and live birth rates following IVF procedures [127]. Additionally, isthmocele can lead to challenges during embryo transfer due to distorted anatomy, particularly in cases of a retroflexed uterus [128].

The presence of isthmocele has been linked to adverse outcomes in IVF treatments, especially in cases of recurrent implantation failure (RIF). Hysteroscopic isthmoplasty has been explored as a potential intervention to improve IVF outcomes in patients with isthmocele and RIF [82]. Furthermore, patients with existing isthmocele face an increased risk of developing intracavitary fluid during hormonal stimulation for IVF, emphasizing the importance of careful monitoring and management during IVF cycles [90].

The impact of isthmocele on IVF success has been a subject of interest, with studies highlighting lower clinical pregnancy rates in women with previous cesarean sections, particularly those with isthmocele [2]. Surgical interventions such as laparoscopic or hysteroscopic isthmocele excision may be considered for patients with significant symptoms, infertility, and failed IVF attempts [129].

It is crucial to address isthmocele in the context of IVF treatment, as it can affect reproductive outcomes and necessitate tailored management strategies. Non-invasive isthmocele treatment options have been proposed as potential pretreatment measures for patients undergoing IVF cycles [109]. Early recognition and appropriate management of isthmocele are essential to optimize IVF success rates and improve overall reproductive outcomes for women with a history of cesarean sections.

The treatment of CSDs is multifaceted and depends on various factors including the age of the patient, the severity of symptoms, associated infertility, residual myometrial thickness (RMT), and the patient’s desire to preserve fertility or the uterus. A comprehensive algorithm for the management of CSDs can be developed based on these considerations [8].

It is important to note that asymptomatic women with CSDs may not necessarily require treatment; however, those who wish to conceive should be carefully evaluated due to the increased risk of complications such as uterine rupture or scar pregnancy. In cases where surgical repair is indicated, the decision between hysteroscopic resection and laparoscopic or vaginal repair should be guided by factors such as RMT.

For patients with RMT > 2.5–3 mm, hysteroscopy appears to be the technique of choice. Particularly, if RMT ≥ 5 mm after the hysteroscopic repair a RMT follow-up during subsequent pregnancy and 39 weeks elective CS is needed.

In cases where the residual tissue is less, recourse to laparotomic, laparoscopic, or vaginal approaches is warranted [130], subsequently, with a post-operative RMT > 3 mm RMT follow-up during a subsequent pregnancy is recommended and elective CS at 39 w is required [8]. With RMT ≥ 3 mm and <5 mm, it is possible to choose a hysteroscopic resection and bottom coagulation; after the repair, if RMT < 3 mm, a laparoscopic or vaginal repair should be discussed, while if RMT follow-up during subsequent pregnancy and 39 weeks elective CS [8].

Both laparoscopic and vaginal repair techniques have shown good anatomical outcomes, with laparoscopy offering the additional benefit of exploring other potential causes of infertility and pelvic pain.

Lastly, in cases where a patient achieves a new pregnancy before the correction of the defect, it is important to consider the mode of delivery. Based on the literature, elective cesarean section before 38 gestational weeks would be recommended to reduce the risk of uterine rupture. The characteristics of the isthmocele should also be considered to optimize surgical technique during cesarean section [2].

## Figures and Tables

**Table 1:** Epidemiology of isthmocele.

Magazine Title	Authors	Year of Publication	Percentage of Patients with Symptoms
Obstetrics & Gynecology	Naji O. et al. [6]	2012	29%
Journal of Minimally Invasive Gynecology	Tulandi T. and Cohen A. [7]	2016	>32%
Fertility and Sterility	Donnez O. [8]	2020	20–70%

**Table 2:** Some studies investigated the possibility of fertility restoration in women with isthmocele with surgical repair. N: number of patients; AUB: abnormal uterine bleeding; IVF: in vitro fertilization; NA: not available; LBR: live birth rate; ECS: elective cesarean section; HR: hysteroscopic repair; VR: vaginal repair; RIF: recurrent implantation failure.

Authors	N	Infertility	IVF pre/without Treatment	Symptoms pre/without Treatment	Technique	Post-Treatment Symptoms	Fertility Post-Treatment	IVF Post-Treatment
Gubbini et al. 2011 [79]	41	3–8 years	0	AUB; pelvic pain	Operative Hysteroscopy	100% resolution	100% pregnancy in 12–24 months; 90.2% ECS (term pregnancy); 9.8% miscarriage/abortion	0
Calzolari et al. 2019 [5]	35	N: 16 1 year after isthmocele diagnosis	0	AUB; pelvic pain	Operative Hysteroscopy	100% resolution	9/16 pregnant (follow up 12–60 months)	NA
Mutlu EA. 2022 [82]	61	N: 61 (with RIF and isthmocele)	N: 30 (LBR 3.3%)	AUB; pelvic pain; RIF	N: 31 Operative Hysteroscopy	NA	spontaneous pregnancy 18.4% (7/38)	n: 31 LBR: 25.8% (8/31)
Cohen SB et al. 2020 [83]	39	N: 21 >1 years. N: 18 > 2 IVF cycles failed	18 (LBR 0%)	AUB and/or pelvic pain or dysuria	Operative Hysteroscopy	79.66% resolution	n° 18 pregnant; n° 2 miscarriage	n° 3 pregnant
Yang G. et al. 2023 [84]	191	N: 70	NA	HR: prolonged menses 96/96; infertility 42/96; niche fluid by US 87/96. VR: prolonged menses 95/95; infertility 28/95; niche fluid by US 93/95	n° 96 = operative Hysteroscopy; n° 95 = vaginal repair	no more niche fluid HR: 61/96; VR 50/95	N° pregnancy wish: 70; HR (39): Clinical Pregnancy 69.2%, LBR 61.5%; VR (24): Clinical Pregnancy 58.3%, LBR 37.5%	Pregnancies: HR = 21/39 (53.8%); VR = 8/24 (33.3%)

## Data Availability

Publicly available datasets were analyzed in this study. These data can be found here: https://pubmed.ncbi.nlm.nih.gov, accessed on 1 February 2024.
